# Human Primary Lens Epithelial Cultures on Basal Laminas Studied by Synchrotron-Based FTIR Microspectroscopy for Understanding Posterior Capsular Opacification

**DOI:** 10.3390/ijms25168858

**Published:** 2024-08-14

**Authors:** Sofija Andjelic, Marko Hawlina

**Affiliations:** Eye Hospital, University Medical Centre, 1000 Ljubljana, Slovenia; marko.hawlina@kclj.si

**Keywords:** primary lens culture, human lens capsule, lens epithelial cells, basal lamina, FTIR, synchrotron light, macromolecular composition, posterior capsular opacification, cataract

## Abstract

Human primary lens epithelial cultures serve as an in vitro model for posterior capsular opacification (PCO) formation. PCO occurs when residual lens epithelial cells (LECs) migrate and proliferate after cataract surgery, differentiating into fibroblastic and lens fiber-like cells. This study aims to show and compare the bio-macromolecular profiles of primary LEC cultures and postoperative lens epithelia LECs on basal laminas (bls), while also analyzing bls and cultured LECs separately. Using synchrotron radiation-based Fourier transform infrared (SR-FTIR) (Bruker, Karlsruhe, Germany) microspectroscopy at the Spanish synchrotron light source ALBA, we observed that the SR-FTIR measurements were predominantly influenced by the strong collagen absorbance of the bls. Cultured LECs on bls showed a higher collagen contribution, indicated by higher vas CH_3_, CH_2_ and CH_3_ wagging and deformation, and the C–N stretching of collagen. In contrast, postoperative LECs on bls showed a higher cell contribution, indicated by the vsym CH_2_ peak and the ratio between vas CH_2_ and vas CH_3_ peaks. The primary difference revealed using SR-FTIR is the greater LEC contribution in spectra recorded from postoperative lens epithelia compared to cultured LECs on bls. IR spectra for bl, cultured LECs and postoperative lens epithelia could be valuable for future research.

## 1. Introduction

The lens capsule or basal lamina (bl) is a transparent and elastic membrane that surrounds the crystalline lens and is of key importance for maintaining the structural integrity of the lens and influencing its transparency. It is a specialized form of extracellular matrix (ECM), with collagen being the most important component. In bls, collagen fibers, particularly collagen type IV, are organized in a mesh-like network [1,2]. The bl provides structural support to lens epithelial cells (LECs) and helps in LECs adhesion, migration and signaling, which are important for the formation of lens fiber cells that fill the inside of the lens. The single-layered lens epithelium, built by LECs, is located in the anterior portion of the lens between the bl and the lens fibers. The average number of LECs is approximately 4000–5000/mm^2^, depending on patient age, with a significant decrease in density in those aged 80 years or older [3,4]. The interaction between LECs and the collagen-rich ECM created by LECs is essential for maintaining the structural and functional integrity of the lens, its transparency and elasticity. However, it is involved also in posterior capsular opacification (PCO) development. PCO occurs secondarily, after cataract surgery. Cataractogenesis, the opacity of the crystalline lens, is the leading cause of blindness, accounting for 48% of all causes of blindness [5]; PCO occurs in approximately 11.8% of cataract surgery cases within the first year, ~20.7% by 3 years and ~28.4% by 5 years [6]. PCO causes quantitative visual disturbances, reduces the quality of vision due to a reduction in contrast sensitivity and is associated with halo effects and a lack of binocular vision [7]. PCO occurs when the remaining LECs, left after cataract surgery in the capsular bag that consists of a part of the anterior and the entire posterior bl [8], start to migrate to the posterior side of the eye. They recolonize denuded regions of the anterior portion of the bl, encroach onto the intraocular lens surface and colonize the previously cell-free posterior bl, ultimately obstructing the visual axis [9,10]. PCO also reflects the proliferation and hypertrophy of residual LECs within the capsular bag [11]. The bl’s residual LECs can trans-differentiate to myofibroblasts, which is important for PCO formation. The myofibroblast cells undergo fibrosis, which results in PCO and blocks light transmission through the lens. It is a complex process that includes morphological transition from epithelial to mesenchymal myofibroblasts (EMT) [12], which is characterized by, among others, the upregulation and secretion of collagens [13]. This upregulation of collagen being involved in EMT was also suggested by the gene expression profiles of pediatric LECs [14]. EMT also generates contractile cells that obstruct the visual axis and give rise to light scatter [15]. LECs being involved in the production of collagenous matrix on bl after the phacoemulsification and aspiration of the crystalline lens and the implantation of the intraocular lens into the capsular bag, resulting in a fibrotic process of the bl, was shown in rabbits [16]. It was immunohistochemically shown that in the cultured LECs derived from human cataract lens epithelia, collagens I, IV, V and VI were present, with strong presentations of types IV and V collagens [17]. Collagens I, V and VI were suggested to be newly produced in the culture, also suggesting that the capsular fibrosis seen after cataract surgery in vivo as a wound healing process of the bl may contain these types of collagens [17]. Nevertheless, although the histopathology of PCO is well characterized, the molecular mechanisms underlying the pathology are still not known [15,18,19,20].

The primary cultures of LECs on bl, obtained from the human lens epithelial explants, can be used as an in vitro model to study PCO formation [21]. We have already studied the structural characteristics of primary cultures of LECs using scanning electron microscopy (SEM) and we also showed their migrating potential and the expression of proliferative and pluripotency specific markers [22], while we have also studied in detail the structural characteristics of intact postoperative human lens epithelium [23]. Moreover, we have studied using SEM and transmission electron microscopy (TEM) the morphology of postoperative lens epithelia obtained from different types of cataracts [24,25,26]. We have also studied using TEM the structure of postoperative bls in cataracts associated with intumescent white cataract and uveitis [27,28].

Structural changes are preceded and/or accompanied by chemical changes. Fourier transform infrared (FTIR) microspectroscopy is a vibrational spectroscopic technique used to analyze the chemical composition of biological samples. The FTIR analysis involves shining infrared light on the sample and measuring the absorption of specific wavelengths. The resulting spectrum provides a fingerprint of the molecular composition of the sample. So far, by using synchrotron radiation-based (SR)-FTIR, we have studied the chemical characteristics and the differences between the postoperative lens epithelia LECs on bls of cortical (C) and nuclear (N) cataracts [29]. Here, we employed SR-FTIR microspectroscopy to understand the bio-macromolecular profiles and the differences between the composites of the primary LECs cultures on bls and postoperative lens epithelia LECs on bls, also showing the fingerprint of bls alone as well as the cultured LECs alone. T our knowledge, SR-FTIR spectroscopy has not been used to study the composites of human primary cultures of LECs on bls until now.

## 2. Results

### 2.1. Differences between the Composites of Cultured LECs on Bls, Cultured LECs Only and Bls Only

**Figure 1:**
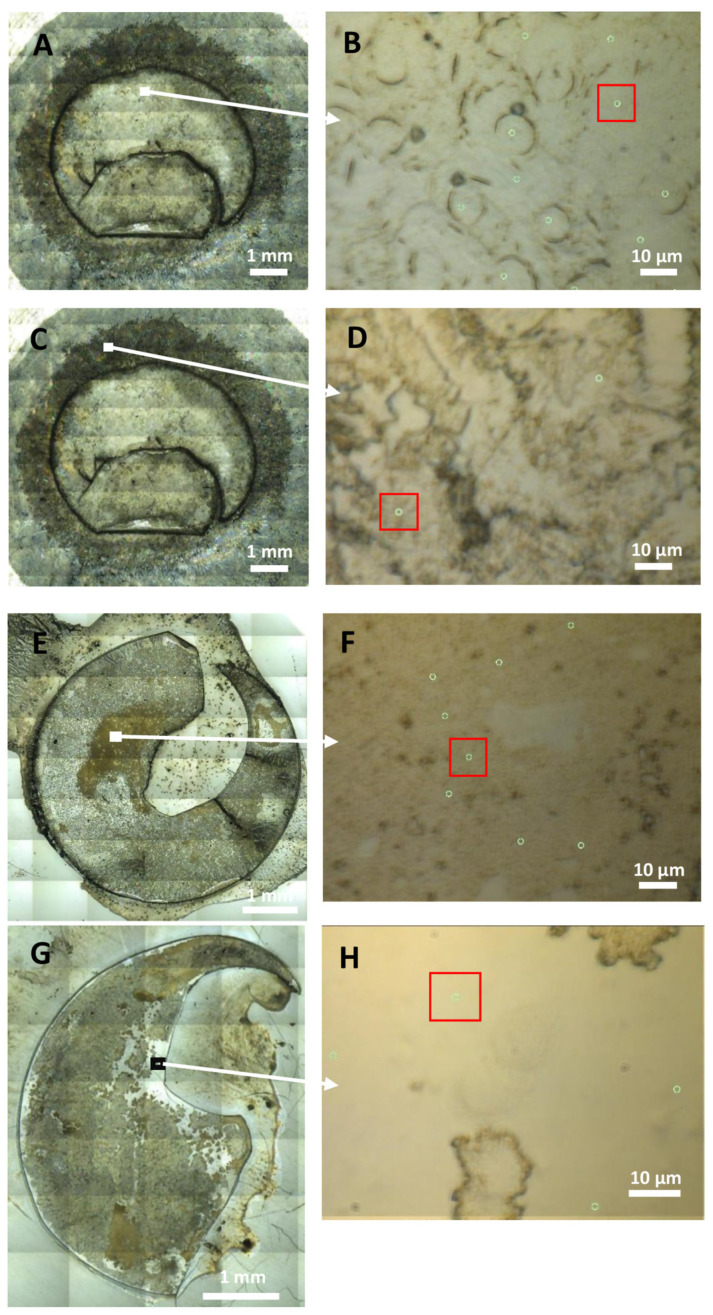
Visible light bright field images of the examples of the following: (**A**,**B**) composites of cultured LECs on bl, (**C**,**D**) cultured LECs only, (**E**,**F**) postoperative lens epithelia LECs on bl and (**G**,**H**) bl only—obtained using reflection geometry (**A**,**C**,**E**,**G**) and transmission geometry (**B**,**D**,**F**,**H**) with enlarged regions showing LECs with a higher magnification, achieved with Schwarzschild objectives; green dots represent the measured locations with red squares representing the size of the measured spots for the spectra used in the analysis.

To distinguish the contribution of bl and cultured LECs on the spectra, we evaluated and compared the spectra of the composites of cultured LECs on bls, cultured LECs only and bls only. The examples of the selected regions are visualized in Figure 1.

**Figure 2:**
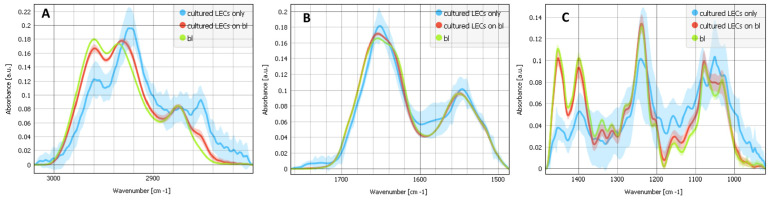
FTIR average spectra with a standard deviation for 3 types of samples: the composites of cultured LECs on bl (red), cultured LECs only (blue) and bls only (green). (**A**) The analysis of the CHx region, attributed to C–H stretching modes (2800–3100 cm^−1^). (**B**) The analysis of the spectral regions of the Amide I, Amide II and carbonyl regions including the ester groups (1485–1765 cm^−1^). (**C**) The analysis of the nucleic acids and carbohydrates spectral region (950–1485 cm^−1^).

Figure 2 shows the SR-FTIR average spectra with a standard deviation for three types of samples: the composites of cultured LECs on bl (red), cultured LECs only (blue) and bl only (green) for all three spectral regions. The analysis of the CHx region (2800–3100 cm^−1^) shows the pronounced absorption band with a maximum at 2852 cm^−1^ in the cultured LECs alone, which correspond to a symmetric CH_2_ vibration, v_sym_ CH_2_, mostly present in cell membranes, while bl alone has no contribution at that wavelength. This indicates that the absorption band with a maximum at 2852 cm^−1^ in the composites of cultured LECs on bl belongs to LECs. The asymmetric CH_2_ vibration, v_as_ CH_2_, at 2925 cm^−1^, also shows the strongest contribution for the cultured LECs alone while the asymmetric CH_3_ vibration, v_as_ CH_3_, at 2960 cm^−1^, shows the strongest contribution for bls alone and the weakest contribution for the cultured LECs alone. The bls alone have a higher v_as_ CH_3_/v_as_ CH_2_ ratio, while the cultured LECs on bls have a lower v_as_ CH_3_/v_as_ CH_2_ ratio. More v_as_ CH_2_ reflects more lipids and more cell membrane. In the protein region (1480–1800 cm^−1^), both for the Amide I and Amide II bands, the SR-FTIR spectra of the composite of the cultured LECs on bl are very similar to the spectra of the bls only. For the cultured LECs only (blue), the Amide I and Amide II bands are shifted to the lower numbers and are sharper as there is much lower collagen contribution, which gives a sharper band. The analysis of the nucleic acids and carbohydrates spectral region (950–1485 cm^−1^) demonstrates that bls alone as well as composites of the cultured LECs on bls show peaks at 1033 cm^−1^ and 1080 cm^−1^, which arise from the C–OH stretching vibrations of the carbohydrate moieties attached to the protein [30,31]. It also shows the absorption features at 1453, 1399, 1339, 1282, 1239 and 1203 cm^−1^ that are attributed to CH_2_ and CH_3_ wagging and deformation, and the C–N stretching of collagen [31,32]. The spectral analysis reveals that bl (green) and cultured LECs on bl (red) show similar bands.

### 2.2. Differences between the Composites of Cultured and Postoperative LECs on Bls

**Figure 3:**
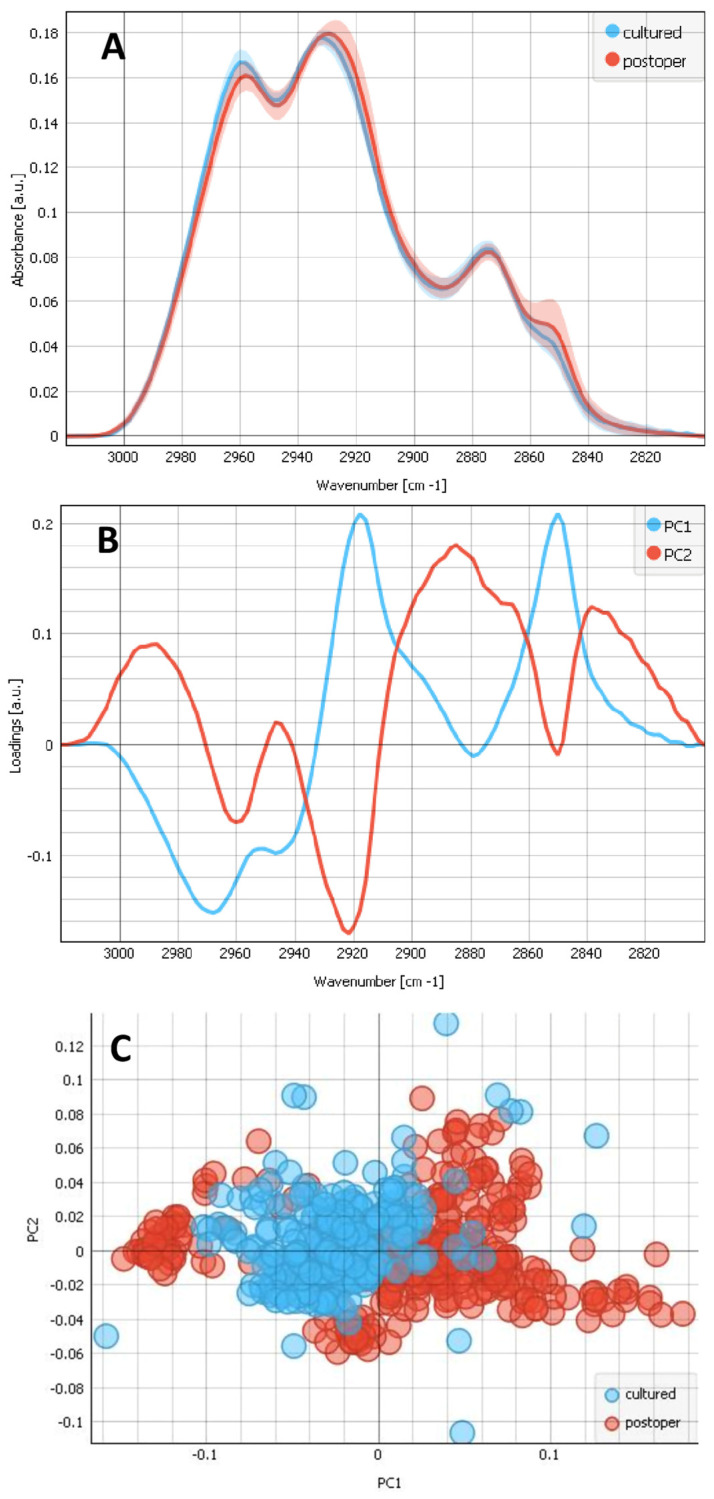
Analysis of the CHx region (2800–3100 cm^−1^), attributed to the C–H stretching modes. (**A**) FTIR average spectra with a standard deviation of 2 types of samples: the composites of cultured LECs on bl (blue) and postoperative lens epithelia LECs on bl (red). (**B**) PCA loadings of the first two components (PC1 in blue and PC2 in red). (**C**) The PCA scores plot denotes the variability associated with the first two components.

We evaluated and compared the spectra of the composites of cultured LECs on bls and the composites of postoperative LECs on bls. The examples of the selected regions are visualized in Figure 1A,E. Figure 3A shows the FTIR average spectra with standard deviations obtained from the CHx region (2800–3100 cm^−1^) for composites of cultured LECs on bls (blue) and the composites of postoperative LECs on bls (red). The average plot shows the pronounced absorption band with maxima for both the composites of cultured LECs on bls and postoperative LECs on bls at ~2960 cm^−1^, which correspond to the v_as_ CH_3_. However, v_as_ CH_3_ shows a stronger contribution for the composites of the cultured LECs on bls than for the composite of the postoperative LECs on bls, indicating the relatively higher collagen contribution of the composites of cultured LECs on bls. From the other side, the most pronounced peak at ~2930 cm^−1^, which corresponds to the v_as_ CH_2_, shows a strong contribution, both in the composites of cultured and postoperative LECs on bls; however, the contribution is stronger for the composites of postoperative LECs on bls. Also, the absorption band with a maximum at 2852 cm^−1^, which reflects LECs, is more present in composites of postoperative LECs on bls, both indicating that the contribution of LECs with respect to the bls is bigger for the postoperative LECs on bls composites. There is another smaller peak on 2875 cm^−1^*,* which corresponds to the symmetric CH_3_ vibration and v_sym_ CH_3_, and is present for both types of composites. This reflects the collagen contribution. A PCA of this CHx region is also shown in Figure 3 with the loadings plots (b) and the PCA score (c). The scores plot shows that the groups separate along PC1 (Figure 3C). The analysis reveals that the two groups (the composites of cultured LECs on bls vs. postoperative LECs on bls) show distinct spectral features, as can be seen in the separation of the two groups in the PCA scores plots. Pronounced changes in the CHx region were observed at 2920 cm^−1^, which correspond to the differences of v_as_ CH_2_ that were more pronounced in the composites of postoperative LECs on bls, reflecting a relatively bigger contribution of LECs with respect to the composite of cultured LECs on bls. PC1 also shows a pronounced band at 2850 cm^−1^, corresponding to the symmetric vibration of CH_2_ and v_sym_ CH_2_, again reflecting the LECs, as it is more present in the composites of the postoperative LECs on bls than in the composites of the cultured LECs on bls.

**Figure 4:**
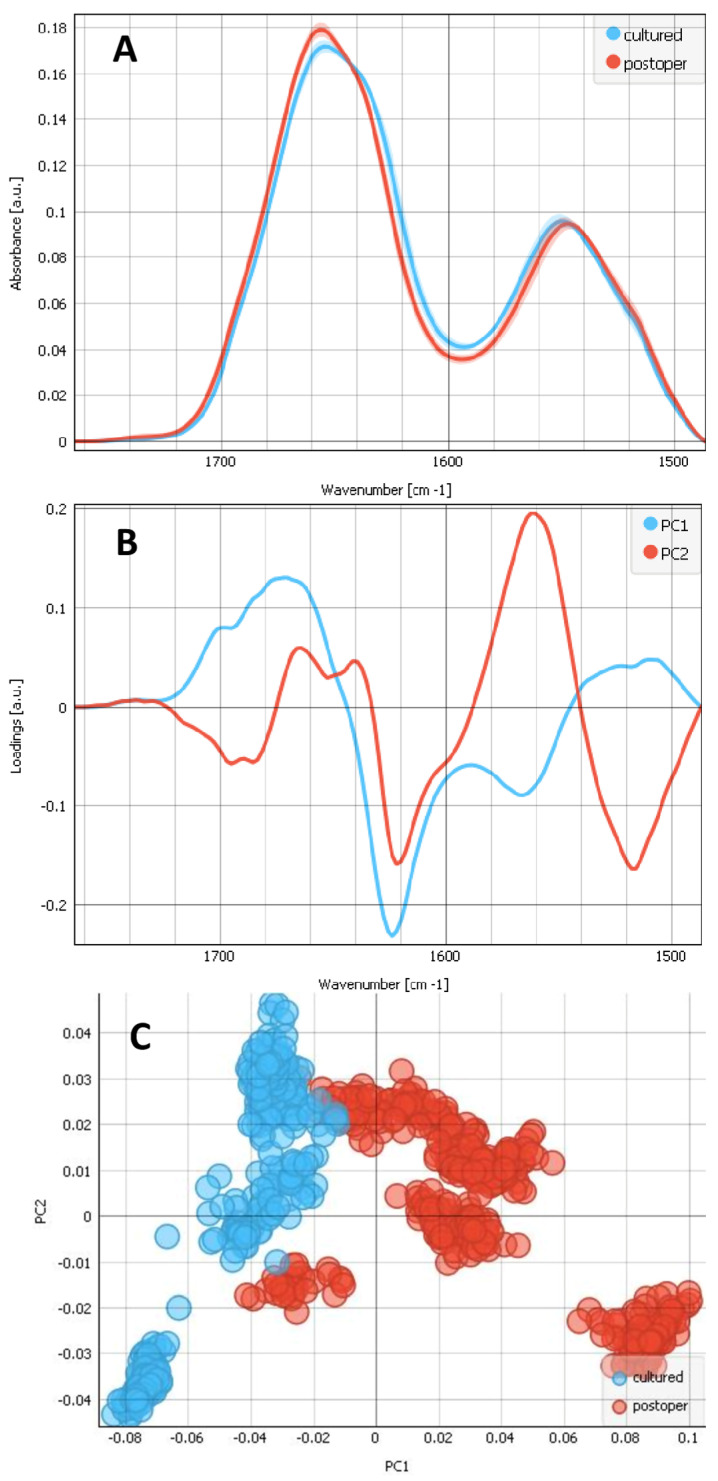
Analysis of the spectral regions of Amide I, Amide II and carbonyl region including the ester groups (1485–1765 cm^−1^) for 2 types of samples: the composites of cultured LECs on bl (blue) and postoperative lens epithelia LECs on bl (red). (**A**) Average FTIR spectra including standard deviation. (**B**) PCA loadings of the first two PCA components (PC1 in blue and PC2 in red). (**C**) The PCA scores plot denotes the variability in the first two components.

The most pronounced parts of the proteins in the Amide I and Amide II regions including the ester groups (1485–1765 cm^−1^) are displayed in Figure 4. The SR-FTIR average spectra with standard deviations are shown in Figure 4A. They show the pronounced absorption bands with two maxima, one at ~1655 cm^−1^ (Amide I) and the other at ~1550 cm^−1^ (Amide II), for both the composites of cultured LECs on bls (blue) and postoperative LECs on bls (red). The Amide I band is shifted to lower wavenumbers for a composite of cultured LECs on bls, with respect to the composite of the postoperative LECs on bls, indicating the higher collagen contribution for the composite of cultured LECs on bls. Amide II band is shifted to higher wavenumbers for a composite of cultured LECs on bls, with respect to the composite of the postoperative LECs on bls, also indicating the higher collagen contribution for the composite of cultured LECs on bls. Petibois et al., 2006, [31], showed collagen’s Amides I and II absorptions at 1658 and 1555 cm^−1^; 1740 cm^−1^ is a sign of oxidation C=O and is more present in postoperative complexes. As the protein bands for Amide I and Amide II are sensitive to changes in protein secondary conformation, these bands were investigated further using PCA. The scores plot shows that the two groups (the composites of cultured LECs on bls (blue) and postoperative LECs on bls (red)) separate along PC1 (Figure 4C).

**Figure 5:**
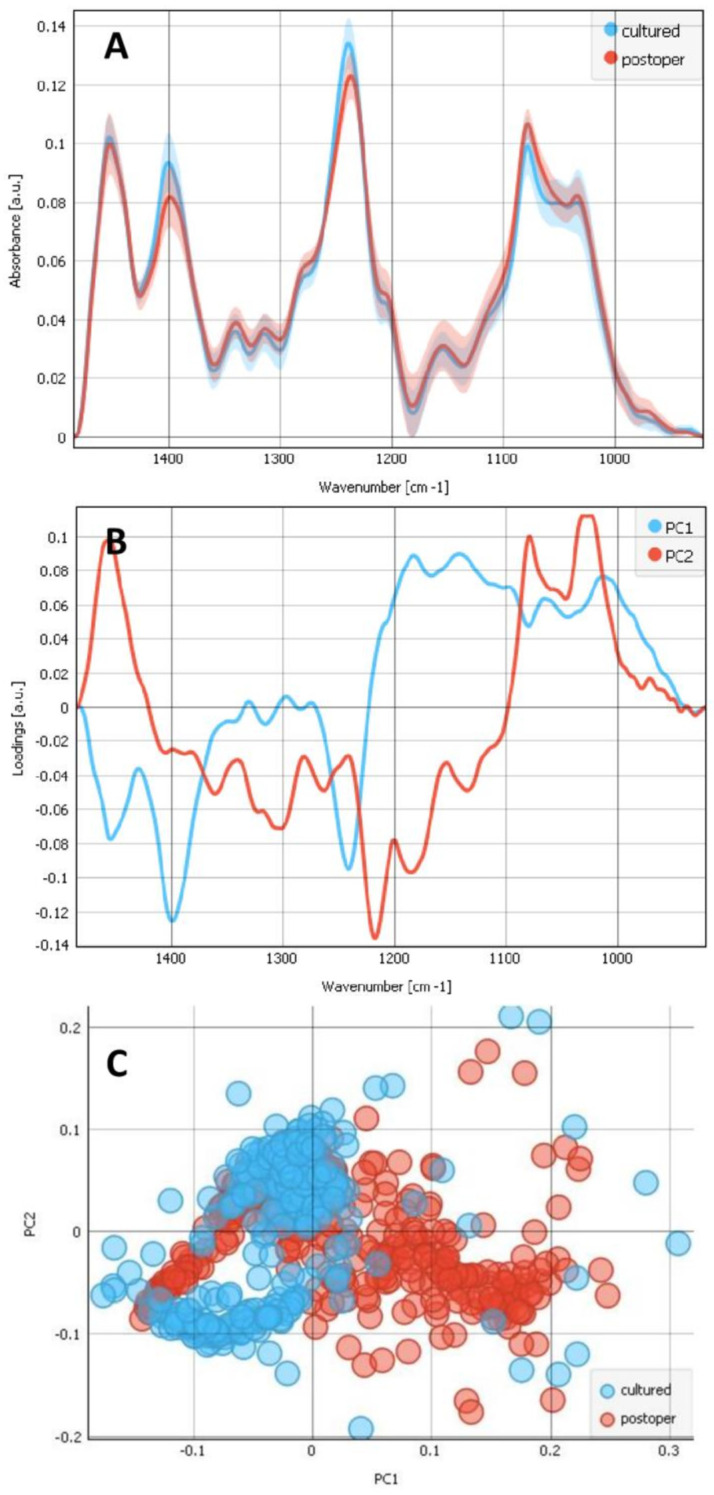
Analysis of the nucleic acids and carbohydrates spectral region (950–1485 cm^−1^). Average spectra (**A**) for 2 types of samples: the composites of cultured LECs on bl (blue) and postoperative lens epithelia LECs on bl (red) and the corresponding loadings plot (**B**) with PC1 (blue) and PC2 (red). The PCA scores (**C**) plot denotes the variability associated with the first two components.

**Figure 6:**
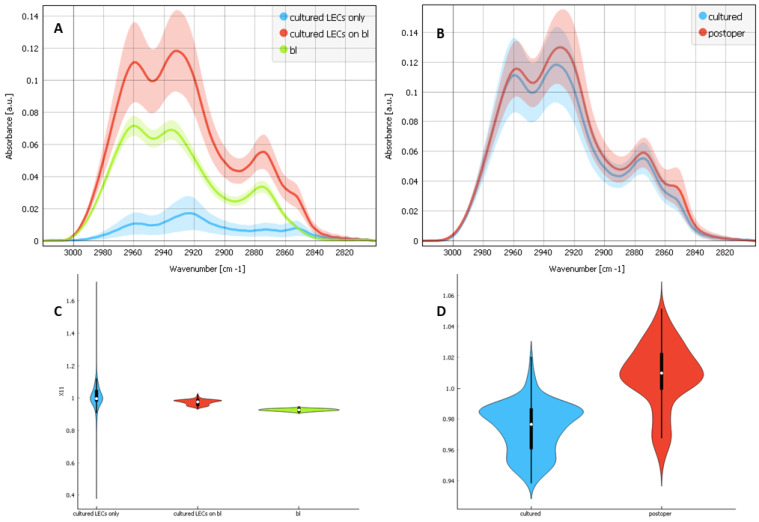
FTIR average spectra with a standard deviation of the CHx region (2800–3100 cm^−1^), attributed to the C–H stretching modes and for (**A**) 3 types of samples: the composites of cultured LECs on bl (red), cultured LECs only (blue) and bls only (green); (**B**) 2 types of samples: the composites of cultured LECs on bl (blue) and postoperative lens epithelia LECs on bl (red). (**C**,**D**) are the ratios between the vas CH2 and vas CH3 peaks for (**A**) and for (**B**), respectively.

The wavenumber region between 950 cm^−1^ and 1485 cm^−1^, corresponding to the nucleic acids and carbohydrates, is shown for the composites of cultured LECs on bls (blue) and composites of postoperative LECs on bls (red) (Figure 5). The average plot (Figure 5A) shows the absorption bands with maxima for both groups, demonstrating that both the composites of cultured LECs on bls and composites of postoperative LECs on bls show absorption features at 1453, 1399, 1339, 1282, 1239 and 1203 cm^−1^ as well as at 1080 cm^−1^ and 1033 cm^−1^. A PCA of this region is also shown in Figure 6, with the loadings plots (b) and the PCA score (c). The scores plot shows that the groups separate weakly along PC1 (Figure 5C). The PC1 loadings plot (Figure 6B) showed a minimum at 1399 cm^−1^ and minimum at 1242 cm^−1^, and a smaller minimum at 1453 cm^−1^, attributed to CH_2_ and CH_3_ wagging and deformation and the C–N stretching of collagen, coming from the bl, being more present in composite of cultured LECs on bls. The analysis reveals that the two groups, the composites of cultured LECs on bls vs postoperative LECs on bls, show the absorption features that come principally from proteins, as shown by Petibois et al., 2006, [31], where type IV and type I collagens exhibited absorptions at 1033, 1059, 1083, 1203, 1236, 1282, 1339, 1399 and 1451 cm^−1^.

### 2.3. Contributions of the Different Components

To check if the cultured LECs on bls have different components contribution than the postoperative lens epithelia LECs on bls, we analyzed (Figure 6) the non-normalized spectra first for the composites of the cultured LECs on bl, cultured LECs only and bls only (Figure 6A), and then for the composites of cultured LECs on bl and postoperative lens epithelia LECs on bl (Figure 6B). We also calculated the ratio between the v_as_ CH_2_ and v_as_ CH_3_ peaks first for the composites of cultured LECs on bl, cultured LECs only and bls only (Figure 6C) and then for the composites of cultured LECs on bl and postoperative lens epithelia LECs on bl (Figure 6D). In the first graph (Figure 6A), the v_as_ CH_2,_ at 2925 cm^−1^ shows the strongest contribution for the cultured LECs alone while the v_as_ CH_3_ at 2960 cm^−1^ shows the strongest contribution for bls alone and the weakest contribution for the cultured LECs alone. More v_as_ CH_2_ reflects more lipids and more cell membrane. The first graph (Figure 6A) also shows that the v_sym_ CH_2_ contribution comes from the cells and that the v_sym_ CH_3_ contribution comes mainly from the bl. The second graph (Figure 6B) shows that in cultured samples there is a lower contribution of LECs (v_as_ CH_2_) and a bigger contribution of bls (v_as_ CH_3_) compared to the postoperative samples, which have a bigger contribution of bls (v_as_ CH_2_) and a smaller contribution of bls (v_as_ CH_3_). Figure 6D also shows that the ratio between the v_as_ CH_2_ and v_as_ CH_3_ peaks is bigger for the composites of postoperative LECs on bls than for the composites of cultured LECs on bls, indicating that the composites of postoperative LECs on bls have bigger LECs and smaller bl contribution. Similarly, the v_sym_ CH_2_ peak at 2852 cm^−1^ (Figure 6B) shows the same, with the postoperative preparations having a bigger contribution of LECs than the cultured preparations. Figure 6C shows that the cultured LECs only have the strongest ratio between the v_as_ CH_2_ and v_as_ CH_3_ peaks, while the bls alone have the smallest; the composites of the cultured LECs on bl have their ratio somewhere between, as is expected.

## 3. Discussion

In this study, we evaluated and compared the bio-macromolecular profiles and the chemical differences between the human composites of primary LECs cultures on bls and composites of postoperative LECs on bls in order to better understand PCO formation. We also showed the fingerprint of the bls alone, as well as the cultured LECs alone. To our knowledge, this is the first time that the SR-FTIR was applied to study the human LECs cultures. In the paper Kreuzer et al., 2020 [29], we compared the postoperative LECs on bls composites from patients with two different cataract types, N and C. Here, we were interested in which way, regardless of the cataract type, postoperative LECs on bls composites differ from the cultured LECs on bls composites. We showed, in parallel, the molecular fingerprint of both the composites of cultured LECs on bl and postoperative lens epithelia LECs on bl. We also confirmed a higher collagen ratio of the cultured LECs on bls composites and a higher cell ratio for the postoperative LECs on bl composites. The peak on 2852 cm^−1^ mainly reflects cell membranes, meaning that the contribution of LECs in respect to the bls is bigger for the postoperative LECs on bl composites. This is also shown by a higher v_as_CH_2_ than v_as_CH_3_ for the cultured LECs alone, both indicating that the composite of the postoperative LECs on bls contains relatively more cell material and less collagen, while the composites of cultured LECs on bls contain relatively more collagen and less cell material.

We showed that bls alone have strong collagen absorbance. However, we do not exclude the potential contribution of other proteins like laminin [2]. In order to discriminate the influence of the spectra of the bl alone, we evaluated and compared the FTIR measurements from the denuded regions of the bls. The regions of denuded bls, without the LECs, were measured on postoperative samples and not on the cultured samples as in the last; due to the migration of the cells, no denuded regions with bl alone could be observed. We previously showed via light microscopy a cell-denuded region of the bl that with culturing becomes recolonized using LECs [22]. The spectra from the bls alone were very much similar to the spectra of pure collagen known from the literature [31]. This suggests also that in the comparison of postoperative lens epithelia from N and C cataracts [29], the N cataract samples had more collagen. It was shown that disruptions in the regulation of collagen production or alterations in the structure of collagen fibers can contribute to cataracts [33]. To see the contribution of the cultured LECs alone, we obtained the SR-FTIR measurements from the confluent cultured LECs grown directly on the CaF_2_ slides. In this way, we could better understand the contributions in the SR-FTIR measurements of the composites of human LECs on bls. To make a comparison that resembles the realistic situation in the lens, with the LECs being attached to the bl in postoperative tissue and migrating on bl during PCO formation and forming fibrous tissue, we compared the composites of postoperative LECs on bls and composites of cultured LECs on bls.

With the SR-FTIR technique, it is not possible to measure the dimensions of the bl and LECs layers from the transversal section. With the additional measuring of the dimensions of bl and LECs layers by some other technique (fluorescent confocal microscopy, TEM), it would be possible to better understand the SR-FTIR signal contributions of bl and LECs, which is what we plan to do in further studies. However, it is known that the bl has regions of variable thickness, with the thickness of the adult human bl between 25 μm and 30 μm at the anterior pole and between 2 μm and 4 μm at the posterior pole [2]. The postoperative lens epithelia’ LECs are, in general, stated to have a typical epithelial morphology. They are regular in shape, usually considered cuboidal, with the dimensions about 5.5–8 μm high and 7–11 μm wide [34] and are tightly packed in a single layer with very little intercellular space. We have shown using TEM that bl is 2–3 × thicker than the preserved attached LECs layer in the human anterior composites of postoperative LECs on bls in different pathologies [24,25]. We have previously provided detailed evidence about their structural organization; by using SEM, TEM and confocal microscopy, we have shown that while the apical side of the LECs oriented toward the fiber cells is smooth, their basal side, which is in contact with the bl, is increased with the extensions and the entanglements of the LECs cytoplasmic membrane [23]. It is possible that these protrusions being involved in the contact between the LECs and bl might also be involved in the LECs migration. In comparison to the postoperative lens epithelia LECs, cultured LECs obtained from the human anterior portion of the bl explants are larger, thinner and irregularly shaped which, as we showed using SEM, depends also on the level of their confluence [22]. New imaging protocols have been developed to image peripheral lens structures using whole mounts of murine ocular lenses [35]. In humans, the whole lens could be obtained from cadavers from the eye bank. Using cadaver lenses for PCO studies would provide valuable insights into the mechanisms underlying PCO. There are many advantages of using human composites of postoperative LECs on bl and the composites of primary cultures of LECs on bls for the studies of PCO. Firstly, the composites of postoperative LECs on bl are regularly excised and normally discarded during cataract surgery, so there is a steady supply of human bl material. Secondly, the experiments are carried out on human cells, which gives a direct connection with human eye pathology helping in understanding it for potential treatment. Regarding animal models, no single animal species is a complete model of the human lens. The composite of postoperative LECs on bl has the advantage of preserving the epithelium in a fairly “intact” configuration, where all, or at least most, of the connections between neighboring LECs and to the underlying bl are preserved, and if the preparation is not mishandled, they behave as they do in their natural environment. Using the cultured human anterior portion of the bl explants and visualizations using light microscopy, SEM and immunofluorescence staining for proliferation and pluripotency markers, we have already shown that the human anterior portion of the bl contains LECs that can migrate and proliferate [22]. This could mimic the process of LECs migration in a capsular bag involved in PCO formation.

We have to stress that the patients from whom we obtained the samples were 70-years-old or older. This is important to bear it in mind, especially considering that PCO is closely associated with patient age and that the aged LECs suppress proliferation and EMT [36].

Understanding the collagen production by LECs is important for gaining insights into the PCO, which shows classic signs of fibrosis: LECs hyperproliferation, migration, deposition of ECM and EMT. While in a healthy lens the collagen-rich ECM created by LECs is essential for maintaining the transparency, elasticity and overall structural integrity of the lens, in PCO, transformed LECs in the posterior bl start producing excessive amounts of ECM components, including collagen [13]. The newly synthesized ECM contributes to the thickening and clouding of the posterior bl. From the other side, SR-FTIR microspectroscopy was shown to be a potent method for the chemical structural characterization of collagen characteristics from various origins, including different natural and synthetic collagens [37]. We therefore suggest the possibility of using SR-FTIR for studying the production by LECs of the ECM components, including collagen, in combinations with fluorescent confocal microscopy.

One of the main questions in the cataract field is how to prevent the PCO remodeling of LECs after surgery. Primary LECs cultures are a model for PCO formation, its prevention and treatment, where we can study how the LECs start to migrate, reconnect, go through EMT and secrete collagens. The prevention of PCO is extensively studied [19]. Many drugs have been studied for their potential treatment effect against PCO [4]. The effort invested in the study of the inhibition of the development of PCO is reflected also in recent works [38,39,40,41].

We hope this study will stimulate the further research of PCO and its potential treatment where SR-FTIR can be applied for the understanding of bio-macromolecular profiles and the chemical differences. Primary LECs cultures can be used to study the drugs affecting PCO formation where inhibiting the growth of LECs is important in preventing the development of PCO. SR-FTIR could be used for the chemical components analysis before and after the treatment. SR-FTIR could also be used to study collagen as a potential adhesive coating or material for IOLs and a scaffold candidate to be incorporated in future tissue engineering-based approaches to address PCO as well as lens regeneration [42]. We hope this work will stimulate further research with the aim of developing possible pharmacological treatments and tissue engineering-based approaches against PCO development.

## 4. Materials and Methods

### 4.1. Tissue Collection and Processing

All tissue collections complied with the Guidelines of the Helsinki Declaration and were approved by the National Medical Ethics Committee of Slovenia, and all patients signed an informed consent form before the operation.

All surgeries were performed at the Eye Hospital, University Medical Centre, Ljubljana, Slovenia. The bls with attached LECs were collected from routine uneventful cataract surgery. The 5–5.5 mm big circles of the central anterior bls were carefully removed through continuous curvilinear capsulorhexis. The bls were dissected so that the anterior portion of the bl and associated LECs were isolated from the fiber cells that form the bulk of the lens. Immediately after isolation, the excised human bls were placed in sterile tubes filled with high-glucose medium (DMEM; Sigma, no. 5671, St. Louis, MO, USA) supplemented with 10% fetal bovine serum (FBS; Gibko, qualified, heat inactivated, origin Brazil) and 1% antibiotics (penicillin–streptomycin; Sigma, no. 4333) and transported to the experimental laboratory of the Eye Hospital.

### 4.2. Primary LECs Cultures

For preparing the primary cultures of LECs on bl, the bls were transferred, one bl specimen per dish, to circular 13 mm × 0.5 mm CaF_2_ slides (Crystan Ltd., Dorset, UK) that were each put in a separate Petri dish. Primary human LECs cultures were established through gently stretching and adherently plating the intact human anterior portion of the bl with LECs on the CaF_2_ slide by using micro-dissecting tweezers (WPI by Dumont, Friedberg, Germany). For obtaining adherent conditions, the careful removal of the remaining medium from the bls was performed with a micropipette, and then viscoelastic (HEALON OVD, Abbott Medical Optics, Santa Ana, CA, USA) was added on top of the bl explant to allow for the flattening or “ironing” of the tissue onto the surface of the CaF_2_ slide [43]. For ex vivo cultivation under adherent conditions, high-glucose medium (DMEM; Sigma, no. 5671, St. Louis, MO, USA) supplemented with 10% FBS (Gibko, qualified, heat inactivated, origin Brazil) and 1% antibiotics (penicillin–streptomycin; Sigma, no. 4333) was then added slowly with the micropipette not to disturb or remove the viscoelastic cover on top of the explants. The micropipette tip was positioned close to the culture dish surface but far away from the bl explant so that the medium arrived softly in contact with the viscoelastic and did not move the explant from its location. The culture dishes were then kept in a CO_2_ incubator (Innova CO-48; New Brunswick Scientific, Edison, NJ, USA) at 37 °C and 5% CO_2_. The culture dish was kept in the incubator without moving for 2–3 days in order to allow the cells to attach and start proliferating out of the bl explant. During medium change, the medium was removed gently and a fresh one was added subsequently with a micropipette from the opposite side of the bl explant in the dish, the pipette tip being close to the surface of the dish all the time. The viscoelastic dissolved over time and was replaced by new medium—the time by which the bl explant was fully attached to the surface of the culture dish. The preparations were cultured until the LECs had recolonized the cell-denuded areas of the bl and had migrated from the bl onto the CaF_2_ slide as observed using light microscopy (Axiovert S100, Carl Zeiss, AG, Oberkochen, Germany). The primary human LEC cultures were, after cultivation, first washed with 5 mL sodium chloride (NaCl, Sigma, St. Louis, MO, USA) for 10 min and then dried under sterile conditions in the laminar flow at room temperature and stored over silica gel prior the measurements at the ALBA synchrotron. The bls explants were from 4 cataract patients: k_1_ bl: 75 years old female, N_2_ cataract, cultured 21 days; k_2_ bl: 77 years old male, N cataract, cultured 14 days; k_3_ bl: 70 years old female, N+C_2_ cataract, cultured 21 days and k_4_ bl: 90 years old female, N+C white cataract, cultured 28 days.

### 4.3. Postoperative Bl with LECs

For preparing postoperative lens epithelia LECs on bl samples, the samples obtained during cataract surgery were first rinsed in 5 mL NaCl (Sigma, St. Louis, MO, USA) for 10 min and then placed by gently stretching and plating adherently on circular 13 mm × 0.5 mm CaF_2_ slides (Crystan Ltd., UK) using micro-dissecting tweezers (WPI byDumont, Med.Biologie, Germany). The samples were dried under sterile conditions in the laminar flow at room temperature and stored over silica gel prior to the measurements at the ALBA synchrotron. The material originated from 9 different cataract patients, with different degrees of cataract development (1: the lowest, 4: the strongest) and with both cataract types: N cataracts: 2xN_1_—36 years old male and 80 years old female, N_2_—68 years old female, 2xN_3_—72 years old male and 84 years old female, N_4_—71 years old male; C cataracts: 2xC_1_—64 and 72 years old females and C_2_—31 years old female. The bigger sample, including here analysed by comparing with the cultured LECs on bls composites, was presented in the article Kreuzer et al., 2020 [29], where it was analyzed in respect to the cataract type.

### 4.4. SR-FTIR Microspectroscopy

In order to assess the organic compounds’ profiles, we performed measurements using the infrared microspectroscopy beamline MIRAS at the ALBA synchrotron light source (Barcelona, Spain) [44]. Conventional FTIR spectroscopy is a useful tool for examining larger cell populations in the tissues. However, the limited brightness of standard infrared light sources generally prevents high spatial (single-cell) resolution measurements in comparison with SR-FTIR microspectroscopy [45]. All SR-FTIR microspectroscopic absorption spectra were collected in transmission mode using the infrared microscope Hyperion 3000 coupled to a Vertex 70 spectrometer (Bruker, Karlsruhe, Germany), equipped with a liquid nitrogen-cooled mercury cadmium telluride detector and the mid-infrared region of the synchrotron light as the infrared light source. Each spectrum was acquired after co-adding 128 scans at a spectral resolution of 4 cm^−1^. We used the OPUS 8.2 (Bruker, Germany) software package for data collection.

In order to achieve the single-cell data acquisition and analysis, we acquired a spectra of 10 × 10 µm^2^ areas of the tissue by using the aperture of the microscope and the highly focused infrared light from the synchrotron source. Visible light bright field images of the following were obtained: composites of cultured LECs on bl (Figure 1A,B), cultured LECs only (Figure 1C,D), postoperative lens epithelia LECs on bl (Figure 1E,F) and bl only (Figure 1G,H); these were obtained in reflection geometry (Figure 1A,C,E,G) and transmission geometry (Figure 1B,D,F,H), the latter also showing the measured locations by the green dots and the size of the measured spots by the red squares. The spectra with an infrared absorbance higher than 2 at the wavenumbers 1650 cm^−1^ and 1020 cm^−1^, were not considered in the analysis. In total, 291 measured individual areas/spectra were analyzed from primary LECs cultures: k1 bl: 50; k2 bl: 102; k3 bl: 50; k4 bl: 89 individual areas/spectra, and in total 425 from postoperative bls, from each 49–50 individual areas/spectra. To distinguish the contribution of bls and cultured LECs on the spectra, the following numbers of measured individual areas/spectra were analyzed in total: 291 from the cultured LECs on bls (k_1_: 50; k_2_: 102; k_3_: 50; k_4_: 89); 286 cultured LECs only (k_1_ LECs and k_3_ LECs: 99 and k_2_ LECs: 88) and 99 bls only (LC_1_: 39 and LC_2_: 60).

The spectral analysis was focused on three regions of the spectra: (1) 950–1485 cm^−1^, i.e., nucleic acids and carbohydrates, (2) Amide I and II (1485–1765 cm^−1^), i.e., proteins and (3) CHx, attributed to the C–H stretching modes (2800–3000 cm^−1^). The spectra were baseline-corrected and the unit vectors normalized in the regions of interest. Data correction and further analysis were performed by using the Quasar 1.3.0 software package (Bioinformatics Laboratory of the University of Ljubljana, Version 3.20.1) with the spectroscopy package [46]. The datasets were compared using a principal component analysis (PCA) and focused on the first two principal components. The spectral data analysis gives qualitative and quantitative data on cell components on the basis of peak shifts, bandwidths and band intensities.

## 5. Conclusions

Primary LEC cultures serve as models to study PCO formation, LEC migration, EMT and collagen secretion. Using human cells provides a direct connection to human pathology, offering better insights for treatment than animal models. The primary difference revealed using SR-FTIR is a higher LECs contribution in spectra recorded from postoperative lens epithelia on bl compared to cultured LECs on bl. IR spectra for bl, cultured LECs and postoperative lens epithelia could be valuable for future research. As primary LEC cultures can be used to test drugs that inhibit LEC growth to prevent PCO, we would like this study to stimulate further research on PCO prevention and treatment, utilizing SR-FTIR to analyze bio-macromolecular profiles and their differences.

## Data Availability

The data presented in this study are available on request from the corresponding author.
